# Bedside ultrasound-guided genicular nerve block with corticosteroids and lignocaine for knee osteoarthritis improves pain and participation, and is safe in inpatient rehabilitation: a retrospective case series

**DOI:** 10.3389/fpain.2025.1601708

**Published:** 2025-05-13

**Authors:** Edmund J. R. Neo, Trier T. N. Lau, Khin Yamin Thein, San San Tay

**Affiliations:** ^1^ Department of Rehabilitation Medicine, Changi General Hospital, Singapore, Singapore; ^2^ Rehabilitation Medicine, SingHealth Residency, Singapore, Singapore

**Keywords:** rehabilitation, knee osteoarthritis, ultrasound, genicular nerve block, pain interventions

## Abstract

**Background:**

Knee osteoarthritis (OA) is a common joint disorder that causes significant pain and disability. It can affect individuals undergoing inpatient rehabilitation, interfering with their participation in therapy and clinical improvement. While there are various treatment options available for this condition, such as the intra-articular corticosteroid injection, not all patients are suitable and symptoms may persist despite multimodal analgesia. The ultrasound-guided genicular nerve block (US GNB) induces analgesia by targeting the genicular nerves around the knee, and has emerged as a safe and effective intervention option. This is the first effort to document its application in the inpatient rehabilitation setting.

**Methods:**

This was a retrospective case series. We reviewed the medical records of inpatients undergoing rehabilitation who underwent the US GNB for disabling knee OA between July 1, 2022, and August 31, 2023. The primary outcome was improvement in rehabilitation participation based on physiotherapist notes in the week following the procedure. Secondary outcomes were pain by visual analogue scale (VAS), ambulation distance, and the Western Ontario and McMaster Universities Arthritis Index (WOMAC), at pre-discharge, 1-month, and 3-month follow-up timepoints. Safety and adverse events were also retrospectively reviewed.

**Results:**

Nine patients were consented for recruitment in our study. Eight of them showed improvement in pain and/or participation in therapy. There were significant improvements in VAS scores (median change -3) and improvements in ambulation distance (median increase 8 m) between pre-injection and pre-discharge phases. However, these did not persist at the longer follow-up visits. There were no serious adverse events although 3 patients had recurrent pain at later dates, and required further procedures or surgical referral.

**Conclusions:**

The US GNB is safe to perform for inpatients undergoing rehabilitation who experience pain from knee OA. We found that in nearly all patients, there was clinical improvement in their pain and participation in therapy. It can be an effective alternative when other analgesia options are less desirable or available, and can help to keep patients progressing on the road to recovery.

## Introduction

Osteoarthritis (OA) is one of the commonest disabling joint disorders, presenting a significant public health challenge, with notable implications on the affected population, healthcare systems, and socioeconomic costs. While many joints can be affected in OA, the knee accounts for 83% of the total OA burden (1).

Knee OA can coexist with other medical conditions such as stroke, hospital-associated deconditioning, and other neuromuscular illnesses. The burden of OA in acute stroke varies but its prevalence is estimated at 6%–52% (2, 3). Recovery from stroke for patients with knee OA undergoing inpatient rehabilitation, for example, can be hindered due to OA-related pain, mobility limitations, and aggravated coping demands. This challenge can be magnified if the remaining unaffected side is the one causing pain. There may also be reduced participation in stroke patients with OA due to analgesic modalities interacting with their stroke treatment (4). It is thus important to promptly manage symptoms from knee OA in patients undergoing inpatient rehabilitation.

Treatments for knee OA include conservative management and surgical treatment such as joint replacement. Up to half of patients still complain of persistent knee pain post-surgery, however (5). In these patients, those who are not surgical candidates, and those who do not want to undergo surgery, the commonest non-operative intervention is the intra-articular corticosteroid injection (IACSI), which has evidence for its symptom-relieving effects (6). The IACSI is not without its risks and contraindications however, such as septic arthritis in patients with intercurrent infections, or the presence of *in situ* hardware from previous surgeries. Patients in inpatient rehabilitation may sometimes decline the IACSI due to previous failed attempts, or concerns regarding other procedural risks, despite experiencing pain during mobilisation and therapy.

Beyond the IACSI, there are a host of other minimally-invasive intervention options for treating difficult knee pain, such as other intra-articular injectates (e.g., platelet-rich plasma and viscosupplementation) (7), genicular artery embolisation (8), and denervation. The genicular nerve block (GNB) is gaining relevance as one such treatment modality (9, 10). The GNB is a type of denervation therapy that was initially used to treat post-operative knee pain. The American Academy of Orthopaedic Surgeons' (AOAS) guidelines suggest that it may reduce pain and improve function in symptomatic knee OA (6). The procedure is typically performed under fluoroscopic guidance and with radiofrequency ablation, but in recent years ultrasound guidance and chemodenervation have gained popularity (11). Though many protocols and nerve targets exist (12), the three most commonly-described nerves are the superior medial genicular nerve (SMGN), superior lateral genicular nerve (SLGN), and inferior medial genicular nerve (IMGN) (13). As the 3-nerve GNB has demonstrated sustained improvements in knee pain and function for up to 6 months (13), patients with stroke and other rehabilitation conditions may be able to benefit from this procedure to relieve their OA-related knee pain, to achieve greater progress in therapy during their stay in the rehabilitation unit.

We have been offering the bedside ultrasound-guided 3-nerve GNB (US GNB) for inpatients who are unable to undergo the IACSI, or declined to do so, over the past 2 years, to good anecdotal effect. Peripheral nerve blocks are a credentialled procedure for our specialty. The US GNB is straightforward to perform, though its use has primarily been in the outpatient setting (13). We intended to conduct a retrospective chart review to objectively evaluate its impact in inpatient rehabilitation.

## Methods

### Aims & hypothesis

The primary aim was to investigate whether bedside US GNB improves participation in rehabilitation for patients with knee OA who were transferred into the inpatient rehabilitation unit. The secondary aim was to report pain, stiffness, and functional outcomes following the US GNB, as well as procedure safety, up to 3 months following the procedure. We hypothesised that the US GNB would improve participation in inpatient rehabilitation as well as outcomes in the medium term, and that it would be safe to perform.

### Study design

The study was conceptualised as a single-site case series performed through medical record review, supported by telephone or face-to-face interviews, for all rehabilitation inpatients who underwent US GNB for knee OA in our acute general hospital, between 1/7/22 and 31/8/23. The study would be reported according to the STROBE guidelines (Strengthening the Reporting of Observational Studies in Epidemiology) (14). Ethics approval was obtained beforehand, as well as senior management endorsement to request an automated database search for patients meeting the case criteria.

Patients with knee pain and a diagnosis of knee OA who were unsuitable for IACSI had been referred to 2 consultants (attending physician-equivalent) for evaluation and consideration of the US GNB. When suitable and clinically indicated, the procedure was carried out. Appropriate written consent had already been obtained out at that point, with comprehensive explanations to patients and decision-making next-of-kin about the background of the procedure, its evidence base, and rationale for recommendation, as well as its novel use in the inpatient rehabilitation setting. For the procedure, our inclusion criteria were: (1) Diagnosis of knee osteoarthritis as evidenced by x-ray or CT findings, of Kellgren-Lawrence grade 2–4; (2) Pain or stiffness limiting function and tolerance/participation with inpatient rehabilitation; (3) Age >21 years old; (4) Able to give informed consent for the procedure (or have a decision-making next-of-kin available to act on their behalf); and (5) English-speaking (either patient or their decision-making next-of-kin). Patients were excluded if they had allergies to any components of the injectate (lignocaine/bupivacaine or triamcinolone), declined the procedure, or were planned for imminent surgical intervention such as arthroplasty or resurfacing.

The conduct of the procedure has been described in a variety of ways (13). We chose to target the SMGN, SLGN, and IMGN only (3-nerve protocol), as they are the most frequently-injected group. Ultrasound guidance with power Doppler was used to locate the superior medial, superior lateral, and inferior medial genicular arteries at the junctions of the epicondyle and femoral or tibial shafts, followed by periarterial injection at the level of the bone cortex (proximal-to-distal in-plane approach for the SMGN and SLGN, distal-to-proximal in-plane approach for the IMGN). We did not target the inferior lateral genicular nerve (ILGN) due to its proximity to the common peroneal nerve and potential for causing inadvertent foot drop. The choice of injectate was 4 mls of 1% lignocaine (40 mg) mixed with 0.5 mls (40 mg) of triamcinolone, divided equally between all 3 sites (1.5 mls each), as they had the best outcomes based on previously-described controlled trials (13).

### Inclusion & exclusion criteria

For this study, the inclusion criteria were:

1. Underwent US GNB for knee pain during the course of their inpatient rehabilitation admission
2. Contactable *via* e-mail or telephone
3. Gave remote or written consent for participation in the study

Suitable patients were mailed a hardcopy letter with an invitation to participate in the study, and subsequently contacted through telephone to seek written consent for their participation, which would take place through a face-to-face visit at their address or a place of their preference. Patients who declined consent would be excluded.

### Outcome measures

The primary outcome measure would be improvement in rehabilitation participation, based on qualitative descriptions reported by the physiotherapists in their clinical documentation, in the week following the intervention. We used a yes/no binary classification to assess the primary outcome measure, defined by whether the patients exhibited less pain, further distance, or longer activity tolerance, through the balance of their inpatient rehabilitation phase. The secondary outcome measures were pain by the single-digit visual analogue scale (VAS), ambulation distance, and the Western Ontario and McMaster Universities Arthritis Index (WOMAC), at the pre-discharge, 1-month-post-discharge, and 3-month-post-discharge timepoints, if they were available. We would also screen their clinical documentation and readmission information for safety-related and adverse events occurring in the following 3 months, such as septic arthritis, haemarthrosis, anaphylaxis, and vascular/systemic injection, among others, as well as progression to knee surgery for worsening symptoms.

We would use routinely-collected information that was recorded as part of the inpatient rehabilitation programme. Pain by VAS is reported by our physiotherapists when patients complain of knee pain during therapy, and their ambulation distance is reported on a daily basis during therapy sessions. We also regularly assess knee pain using the WOMAC which is a widely-used standardised questionnaire comprising 24 questions about pain, stiffness, and functional capabilities, for patients with hip and knee pain (15). The WOMAC is well-studied with good validity and reliability, and our clinical teams use it as a standard language for evaluating patient-reported severity of knee dysfunction.

Other reportable information that we would collect included demographic and anthropometric data (age, gender, weight, height, body mass index), duration of symptoms, baseline VAS/WOMAC scores, baseline ambulation capability, and radiological severity.

### Statistical analysis

Descriptive statistics would be used to evaluate measures of central tendency for demographic and anthropometric data. Though the numbers would not be sufficient to power conclusions on efficacy/effectiveness, basic inferential statistics (paired *t*-test for parametric data, and Wilcoxon signed-rank test for non-parametric data) would be performed for other secondary outcome measures such as VAS and WOMAC. We would use IBM SPSS version 23.0 (IBM Corp, Armonk, NY, USA) for statistical analysis, with a *p*-value < 0.05 considered to be statistically significant. Missing data that could not be recovered through the chart review or patient recollections would be declared as-is.

Sample size and power calculation were not performed as this was a retrospective chart review/case series. Data accumulated (estimated group size 10–20) would thus not be of a quantity or quality to inform inferential conclusions about efficacy/effectiveness.

## Results

**Figure 1:**
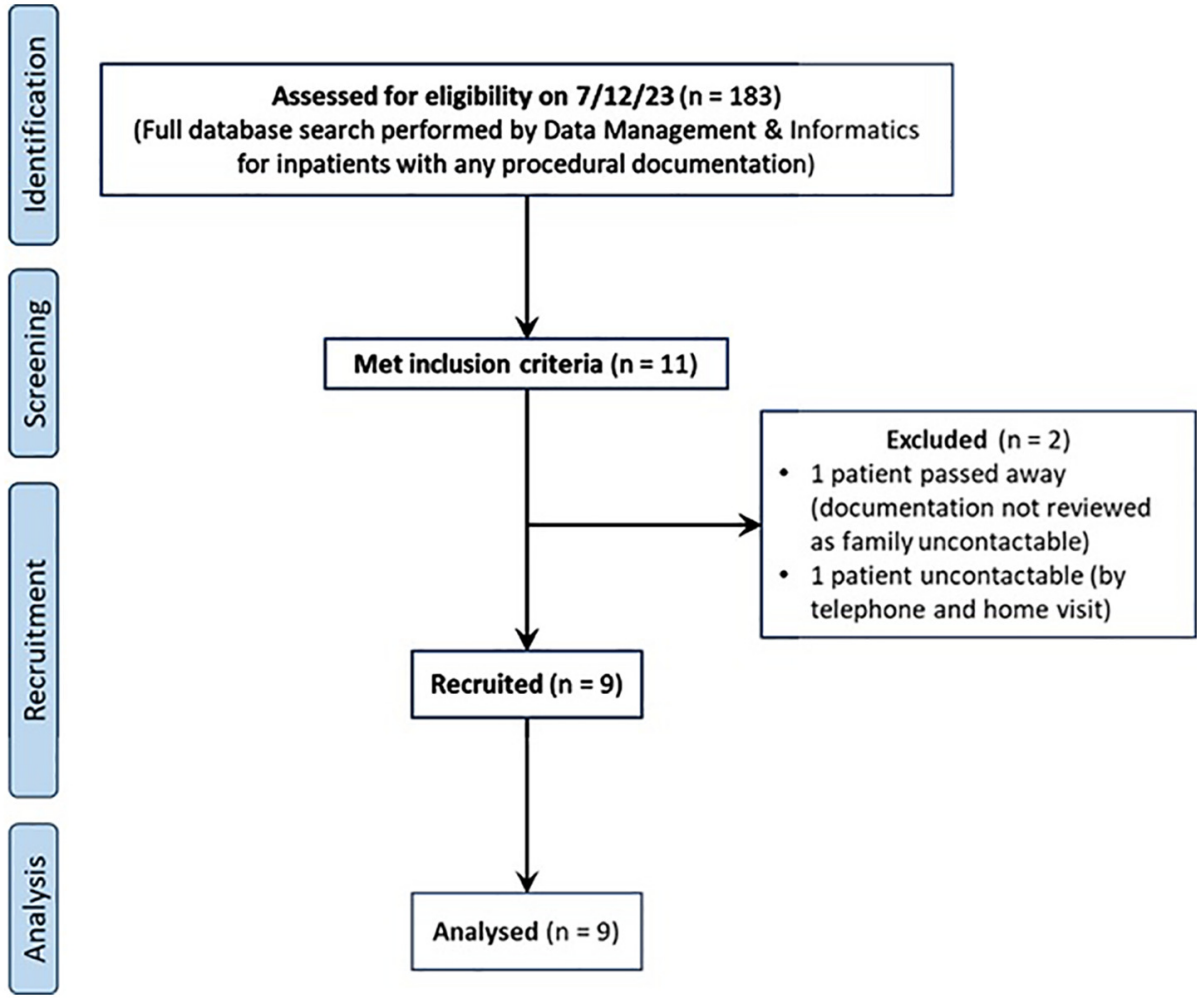
Flow diagram showing screening and recruitment process.

A database search performed on 7/12/23 returned 183 inpatients with any procedural documentation. Of these, 11 unique patients met the inclusion criteria and were approached for recruitment. 1 patient had passed away in the previous year and her records were not accessed as there was no contactable next-of-kin or legal representative available. Another patient was uncontactable despite repeated phone calls and a home visit. The rest of the eligible patients (9 total) provided consent for participation (Figure 1).

### Demographics & clinical baseline

Of the patients who provided consent for inclusion, the average age was 71.2 years (±.8) (Table 1). 8 patients (89%) were male. Median body mass index (BMI) was 18.4 (interquartile range 19.9–31.0). All patients had a prior diagnosis of knee OA, of radiological grade 2 in 3 patients, grade 3 in 3 patients, and grade 4 in 2 patients. The last patient had hardware present in his symptomatic knee and was not graded. There was unilateral pain in 6 patients (2 right, 4 left) and bilateral pain in 3 patients. Symptom duration was 0–5 years in 4 patients, 5–10 years in 1 patient, and >10 years in 1 patient (3 were unable to recall).

**Table 1:** Patient demographics.

Demographics		*n* = 9
Age, mean yrs (SD)		71.2 (±8.8)
Gender (%)	Male	8 (88%)
	Female	1 (11%)
Weight, kg (IQR)		59.2 (49.8, 71.4)
BMI, median (IQR)		18.4 (19.9, 31.0)
Cause of knee pain (%)		Osteoarthritis (100%)
Affected side (%)	Left	4 (44%)
	Right	2 (22%)
	Both (more symptomatic side was injected)	3 (33%)
Kellgren-Lawrence grade (%)	1	0 (0%)
	2	3 (33%)
	3	3 (33%)
	4	2 (22%)
	Not applicable (hardware present)	1 (11%)
Symptom duration (%)	0–5 yrs	4 (44%)
	5–10 yrs	1 (11%)
	>10 yrs	1 (11%)
	Unknown/unable to recall (with no prior x-rays)	3 (33%)
Admitting condition (%)	Stroke affecting ipsilateral side	1 (11%)
	Stroke affecting contralateral side	2 (22%)
	Other neurological conditions	1 (11%)
	Musculoskeletal conditions including deconditioning	5 (55%)
Reason for undergoing GNB (%)	Failed IACSI	2 (22%)
	Declined IACSI	1 (11%)
	Ongoing sepsis	4 (44%)
	Immunosuppressed	1 (11%)
	Hardware in-situ	1 (11%)
Analgesia usage (%)	Paracetamol	9 (100%)
	NSAIDs/COX-2 inhibitors	0 (0%)
	Weak opioids	7 (78%)
	Strong opioids	1 (11%)
	Topical analgesia	6 (67%)
	Other adjuvants	1 (11%)
Baseline ambulation distance, m (pre-injection, in-hospital, %)	Unable	2 (22%)
	0–20	6 (67%)
	20–200	1 (11%)
	>200	0 (0%)
Baseline VAS, median (pre-injection, in-hospital) IQR)		4 (0.5, 6)
Baseline WOMAC, median (pre-injection, in-hospital, IQR)	Pain subscale	6 (3.8, 11)
	Stiffness subscale	2 (0, 5.5)
	Function subscale	28.5 (10.8, 34.5)

The admitting condition was “stroke or other neurological conditions” in 4 patients, and “musculoskeletal conditions (including deconditioning)” for the other 5. All patients were prescribed paracetamol, with 7 on weak opioids, 6 using topical analgesia, 1 using strong opioids, and 1 on adjuvant analgesia. The median VAS was 4 (IQR 0.5–6), and most patients (6) were only able to ambulate for 0–20 m. 2 patients were unable to ambulate (both had stroke on the contralateral side), and 1 was able to ambulate >20 m. Median WOMAC scores by subscale were: pain 6 (IQR 3.8–11), stiffness 2 (IQR 0–5.5), and function 28.5 (IQR 10.8–34.5). Reasons for agreeing to undergo the GNB were: failed IACSI (2 patients), declined IACSI (1), ongoing sepsis (4), immunosuppressed (1), and hardware *in situ* (1).

### Post-injection results

The procedure was performed uneventfully in all patients. 1 patient however had a suprapatellar effusion that was observed during the pre-procedure localisation scan, which required procedural modification—in this patient the SMGN and SLGN were traced further medially and laterally respectively, and the needle's approach was from a posterior-oblique direction, with care taken to avoid penetrating the suprapatellar bursa.

**Figure 2:**
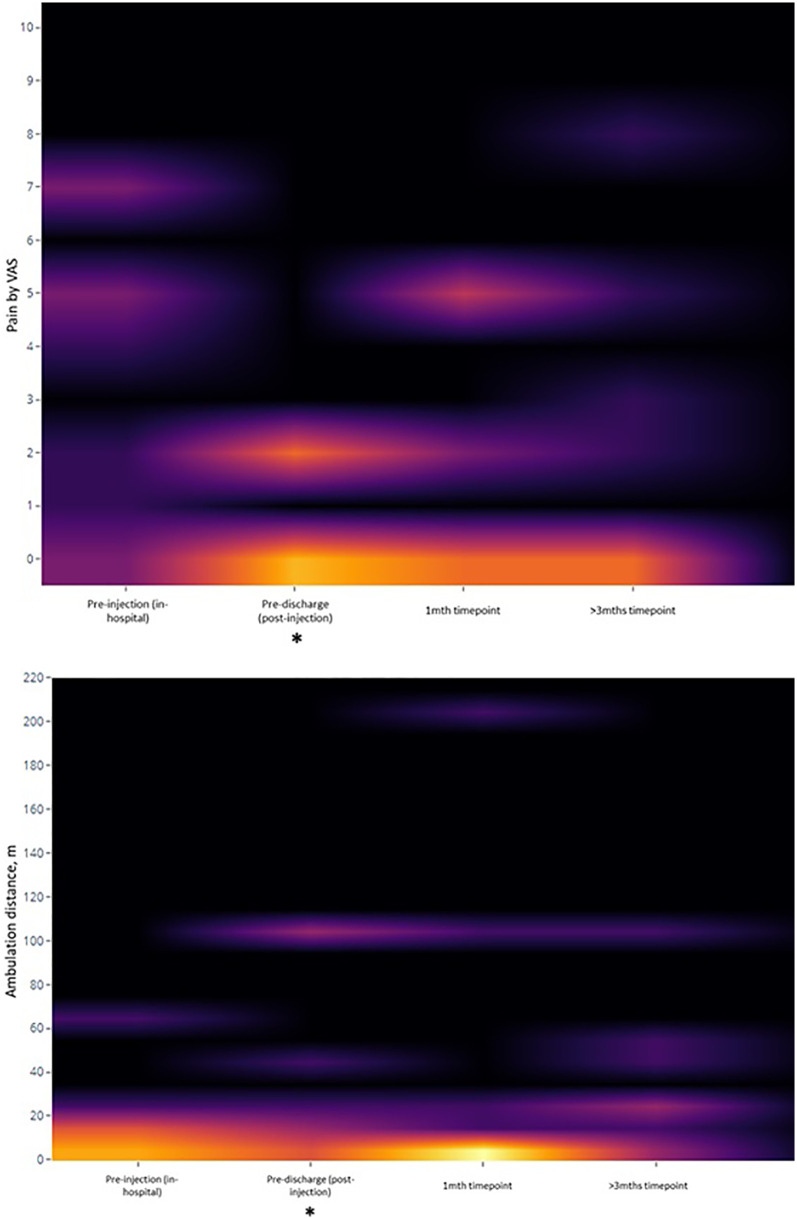
Heatmap of key secondary outcomes over time. *: statistically-significant change from baseline value.

After the procedure, 1 patient experienced an immediate increase in pain while the rest had reduced or no pain. 8 of the 9 patients were considered to have improved, with pain reduction by VAS and/or improved ambulation distance that was documented by the therapists and maintained across the balance of their inpatient stays (of varying durations) (Table 2). The final patient had persistent pain that continued to impede his participation in therapy. Taken as a group, there were significant improvements in VAS (median change -3, *p* = 0.042) and ambulation distance (median increase 8 m, *p* = 0.018) between the pre-injection and pre-discharge phases (Figure 2), though the WOMAC subscale scores did not change significantly during the same period.

**Table 2:** Primary as well as key secondary outcomes, comparing the pre-injection and pre-discharge phases.

S/No	Admitting condition	Qualitative report by therapists	Considered as improvement?	Pre-injection (in-hospital)		Pre-discharge (post-injection)	
				VAS	Ambulation distance	VAS	Ambulation distance
1	Stroke affecting ipsilateral side	Immediately more painful, but went down to VAS 0 for remainder of stay; more participative and even went for exercise class	Yes	7	15 m	0	15 m
2	Stroke affecting contralateral side	Lasted 3 days, immediately able to walk, but pain gradually recurred during hospitalisation	No	0	0 m	0	5 m
3	Stroke affecting contralateral side	Pain gone by 4th day, and remained pain-free through balance of subacute rehab decantment (>1 mth)	Yes	5	0 m	0	8 m
4	Other neurological conditions	Remained in pain until day of discharge, but able to walk slightly more (7 m) later on while in a step-down rehabilitation facility	Yes	0	5 m	2	5 m
5	Musculoskeletal conditions including deconditioning	Immediately less painful and disappeared by 4th day, but gradually recurred (mild and tolerable) throughout balance of stay	Yes	7	20 m	2	40 m
6	Musculoskeletal conditions including deconditioning	Walked 200 m (20x more) the next day, pain-free through the balance of admission (1 more week)	Yes	4	10 m	0	100 m
7	Musculoskeletal conditions including deconditioning	Pain decreased and was totally gone by D7, remained pain-free through rest of admission	Yes	5	10 m	2	20 m
8	Musculoskeletal conditions including deconditioning	No pain reported on the next day, could walk 100 m without breaks, stable across rest of stay	Yes	2	60 m	0	100 m
9	Musculoskeletal conditions including deconditioning	Pain improved, able to participate in PT sessions across balance of hospitalisation (12 days)	Yes	1	5 m	2	10 m

At the 1-month and 3-month post-procedure follow-up periods, however, there were no significant changes from the pre-injection baseline for any of the VAS, ambulation, or WOMAC parameters. Using analysis of covariants (ANCOVA), Kellgren-Lawrence grades and sidedness of symptoms (unilateral or bilateral) were not predictive of changes in pain or ambulation distance at any timepoint. Only symptom duration (in months) was predictive of changes in VAS between pre-injection and pre-discharge [ΔVAS = 0.961–0.54 (duration), *p* = 0.007], as well as between pre-injection and 3 months [ΔVAS = 2.065–0.57 (duration), *p* = 0.047], following simple linear regression.

### Safety & adverse events

No serious adverse events were reported for any patient. However, 3 patients reported disabling recurrence of the pain at follow-up—2 were offered repeat injections with dehydrated alcohol/lignocaine mix, and 1 was referred to Orthopaedic Surgery for consideration of arthroplasty.

## Discussion

### Prompt pain control in patients undergoing rehabilitation

OA is associated with longer in-hospital length-of-stay (LoS) as well as slower long-term recovery (3), and knee OA has a negative impact on the recovery of functional ambulation post-stroke (16). Although delays in rehabilitation arising from the pain and disability caused by knee OA are not entirely avoidable, the knock-on effects on neuroplasticity during the “golden window of recovery” (in stroke, among other neurological disorders) can be detrimental to patients (17). Prompt treatment would allow patients to maintain the tempo of their recovery, and we often give oral and topical analgesia as indicated. When patients' pain persists and initial multimodal analgesia remains insufficient, we have found the bedside US GNB to be a useful alternative when performing the IACSI is not ideal. Within our unit, where the average LoS for inpatient rehabilitation is 2 weeks, and there is a high demand for beds, every rehabilitation touchpoint is crucial, and the US GNB thus allows us to maximise the volume of therapy delivered.

### Impact of the GNB on rehabilitation inpatients

Performing the GNB allowed the two non-ambulant stroke patients (who had post-stroke paresis on one side and knee pain on the other) to begin gait training thereafter, upgrading their Functional Ambulation Category scores from 0 for both, to 1 and 2 respectively. We considered this to be a very significant change as the patients could then participate more intensively in therapy. Neurological disorders such as stroke, spinal cord dysfunction, and parkinsonism can be severely disabling and even a slight change in mobility status can impact massively on patients' therapy and mobilisation options. Even for other rehabilitation conditions such as hospital-associated deconditioning, interference with therapy and self-directed exercise due to bed rest can interrupt and undo strength and functional gains (18). With missed therapy from pain, there are also downstream effects on rehabilitation efficiency, LoS, discharge goals, and costs of hospitalisation. Having another interventional pain option for our inpatients that is safe and impactful is thus sensible at a systemic level.

### Technical aspects of the US GNB

Compared to other options for managing knee OA in rehabilitation patients, such as oral analgesia, topical analgesia, non-medical adjuvants, and injectables like the IACSI or viscosupplementation, the US GNB is considerably more complex. It requires 3 punctures (in our approach, though more comprehensive regimens have been described) (13), with the associated puncture-related discomfort, and anatomical variation can make sonographic localisation of the genicular neurovascular bundles challenging, leading to a prolonged pre-procedure study. The coexistence of a suprapatellar effusion in some patients also makes targeting of the SMGN and SLGN a challenge. The suprapatellar recess frequently communicates with the knee joint (19), and with a low-yet-possible risk of bacterial septic arthritis following intra-articular corticosteroid injections (20), puncturing the effusion twice during the US GNB is undesirable. We suggest to trace the neurovascular bundles posteriorly until the effusion is not visible in the probe's field of view, or approach obliquely from as posterior as possible, to reduce the chance of inadvertent suprapatellar puncture. This is especially so if ongoing sepsis or immunocompromise are factors in the choice of this procedure over the IACSI. Proper sterile technique is a good habit, regardless of whether there is a suprapatellar effusion, due to the nerves' proximity to the knee joint.

Other technical factors include the need for pre-procedure/intra-procedure analgesia as peri-osseous deposition of fluid can be painful, similar to other genicular nerve procedures such as cryoneurolysis (21). Patients with larger habitus may require the use of a lower-frequency probe or out-of-plane approach to ensure that the needle tip is able to reach the genicular nerves, in particular the SMGN and SLGN, which was our experience.

An intrinsic limitation of our intervention approach was the decision to proceed with a 3-nerve protocol when more complex regimens exist. We sought a balance between analgesic coverage and number of punctures. The suggestion of the recurrent fibular nerve as a potential lesioning alternative to the ILGN is promising (12), and newly-proposed updates in the sonoanatomic localisation of the SMGN and SLGN confer implications for future procedural studies (12).

### Long-term maintenance of outcomes

Surprisingly, there was no significant maintenance of gains made in the outpatient follow-up phase, be it for pain, mobility, or other outcomes. This contradicts other randomised controlled trials which had used a corticosteroid/lignocaine mix and reported significant improvements up to at least 3 months (22–24). We postulate that patient-specific factors like the higher acute comorbidity burden (knee OA + another disabling condition), or procedure-specific factors like the avoidance of the ILGN, may be responsible for this difference. In our unit however, this lack of longer-term efficacy has driven a shift in injectate selection, and we have now started to use dehydrated alcohol instead of corticosteroids for its longer-term neuroablative effect, which has been reported to last for 6 months (25). The differences in mechanism and duration of action would make alcohol genicular nerve neurolysis (EtOH-GNB) a sensible alternative (26), and we note the similar adoption of phenol in other units for this procedure (27). Considerations should include the risk of dysesthesias as a side effect (27), and in our inpatients, the risks of nosocomial and iatrogenic sepsis from the ward environment and procedure respectively.

### Limitations

The main limitation of our findings is the small available sample size and retrospective non-controlled study design, which demands cautious interpretation of our statistical inferences. There was also a 19.4% data loss for the follow-up WOMAC outcomes (as some of these had not been documented, or patients were unable to recall their function at these timepoints). This could be due to the complexity of the outcome measure, which measures functional aspects that may not necessarily change in a short duration.

Where the US GNB should fall in a patient management algorithm for knee OA also remains unclear, with multiple other clinical considerations in this studied population (patients with acute stroke cannot undergo arthroplasty immediately; patients with active sepsis are unsuitable for corticosteroid injection), though their cases reflect the complexity of real-world challenges in inpatient rehabilitation.

## Conclusion

Knee OA in inpatient rehabilitation can disrupt participation and patient progress. To date, this is the first study of the application of US GNB for rehabilitation inpatients with this condition. While the US GNB is safe to perform, and improved pain and participation in therapy in the short-term, these benefits unfortunately did not persist beyond discharge. Despite that, it gave us an important and reliable adjunct for our patients' analgesia, that was effective for their inpatient rehabilitation phase. Further work in this population could help to improve the potential of this procedure, giving us more options to support patients as they journey to recovery.

## Data Availability

The original contributions presented in the study are included in the article/Supplementary Material, further inquiries can be directed to the corresponding author.
